# Association of Playing College American Football With Long-term Health Outcomes and Mortality

**DOI:** 10.1001/jamanetworkopen.2022.8775

**Published:** 2022-04-20

**Authors:** Alyssa Phelps, Michael L. Alosco, Zachary Baucom, Kaitlin Hartlage, Joseph N. Palmisano, Jennifer Weuve, Jesse Mez, Yorghos Tripodis, Robert A. Stern

**Affiliations:** 1 Boston University Alzheimer’s Disease Research Center and CTE Center, Boston University School of Medicine, Boston, Massachusetts; 2 Department of Neurology, Boston University School of Medicine, Boston, Massachusetts; 3 Department of Biostatistics, Boston University School of Public Health, Boston, Massachusetts; 4 Biostatistics and Epidemiology Data Analytics Center, Boston University School of Public Health, Boston, Massachusetts; 5 Department of Epidemiology, Boston University School of Public Health, Boston, Massachusetts; 6 Department of Neurosurgery, Boston University School of Medicine, Boston, Massachusetts; 7 Department of Anatomy & Neurobiology, Boston University School of Medicine, Boston, Massachusetts

## Abstract

**Importance:**

Exposure to repetitive head impacts from playing American football (including impacts resulting in symptomatic concussions and subconcussive trauma) is associated with increased risk for later-life health problems, including cognitive and neuropsychiatric decline and neurodegenerative disease. Most research on long-term health consequences of playing football has focused on former professional athletes, with limited studies of former college players.

**Objectives:**

To estimate the prevalence of self-reported health conditions among former college football players compared with a sample of men in the general population as well as standardized mortality ratios (SMRs) among former college football players.

**Design, Setting, and Participants:**

This cohort study included data from 447 former University of Notre Dame (ND) football players aged 59 to 75 years who were seniors on the rosters from 1964 to 1980. A health outcomes survey was distributed to living players and next of kin of deceased players for whom contact information was available. The survey was completed from December 2018 to May 2019.

**Exposure:**

Participation in football at ND.

**Main Outcomes and Measures:**

Prevalence of health outcomes was compared between living former players who completed the survey and propensity score–matched participants in the Health and Retirement Study (HRS). Standardized mortality ratios of all causes and specific causes of death among all former players were compared with those among men in the general US population.

**Results:**

A total of 216 living players completed the health survey (median age, 67 years; IQR, 63-70 years) and were compared with 638 participants in the HRS (median age, 66 years; IQR, 63-70 years). Former players reported a higher prevalence of cognitive impairment (10 [5%] vs 8 [1%]; *P* = .02), headaches (22 [10%] vs 22 [4%]; *P* = .001), cardiovascular disease (70 [33%] vs 128 [20%]; *P* = .001), hypercholesterolemia (111 [52%] vs 182 [29%]; *P* = .001), and alcohol use (185 [86%] vs 489 [77%]; *P* = .02) and a lower prevalence of diabetes (24 [11%] vs 146 [23%]; *P* = .001). All-cause mortality (SMR, 0.54; 95% CI, 0.42-0.67) and mortality from heart (SMR, 0.64; 95% CI, 0.39-0.99), circulatory (SMR, 0.23; 95% CI, 0.03-0.83), respiratory (SMR, 0.13; 95% CI, 0.00-0.70), and digestive system (SMR, 0.13; 95% CI, 0.00-0.74) disorders; lung cancer (SMR, 0.26; 95% CI, 0.05-0.77); and violence (SMR, 0.10; 95% CI, 0.00-0.58) were significantly lower in the ND cohort than in the general population. Mortality from brain and other nervous system cancers was significantly higher in the ND cohort (SMR, 3.82; 95% CI, 1.04-9.77). Whereas point estimates were greater for all neurodegenerative causes (SMR, 1.42; 95% CI, 0.29-4.18), amyotrophic lateral sclerosis (SMR, 2.93; 95% CI, 0.36-10.59), and Parkinson disease (SMR, 2.07; 95% CI, 0.05-11.55), the difference did not reach statistical significance.

**Conclusions and Relevance:**

In this cohort study of former college football players, both positive and negative health outcomes were observed. With more than 800 000 former college players living in the US, additional research appears to be needed to provide stakeholders with guidance to maximize factors that improve health outcomes and minimize factors that may increase risk for later-life morbidity and mortality.

## Key Points

**Question:**

What were the prevalence of self-reported health conditions and mortality rate in a cohort of older former college American football players?

**Findings:**

In this cohort study of 447 former college football players aged 59 to 75 years, compared with a matched sample of men in the general population, former living players who completed a survey had a significantly higher prevalence of cognitive impairment disorders, recurrent headaches, cardiovascular disease, and hypercholesterolemia but a lower prevalence of diabetes. Although overall mortality among all former players was significantly lower than that in the general US male population, mortality from brain and other nervous system cancers was higher.

**Meaning:**

The findings suggest that additional research is needed to provide stakeholders with guidance to maximize factors that improve health outcomes and eliminate or reduce factors that may increase risk for later-life morbidity and mortality.

## Teaser

This cohort study compares the prevalence of self-reported health conditions among former college football players with that in a sample of men in the general population as well as standardized mortality ratios among the former college football players with those in the general US population of men.

## Introduction

There has been increasing interest in later-life health outcomes among professional American football players. In particular, research has shown that exposure to repetitive head impacts (RHIs) from football, including RHIs resulting in symptomatic concussions and the more common subconcussive trauma, is associated with worse later-life cognitive^1,2,3,4^ and neuropsychiatric function,^5,6,7^ fluid and neuroimaging evidence of neurodegeneration,^8,9,10,11,12,13^ and risk for postmortem diagnosis of neurodegenerative diseases, including chronic traumatic encephalopathy^14,15,16,17^ and amyotrophic lateral sclerosis (ALS).^18,19^ Professional football players are also at increased risk for sleep apnea,^20^ rapid-eye-movement sleep behavior disorder,^21^ cardiovascular disease (CVD),^22^ atrial fibrillation,^23^ and opioid use.^24^ Despite the higher prevalence of CVD during life, mortality rate studies suggest that former National Football League (NFL) players live longer and have lower CVD mortality compared with men in the general US population, possibly because of greater physical activity and less cigarette smoking.^25,26,27^ In contrast, former NFL players have been reported to have a greater risk of death from neurodegenerative causes, including ALS and Alzheimer disease.^28,29^ Lehman and colleagues^29^ examined mortality rates among 3439 former NFL players who played between 1959 and 1988 and found that although the former players had an overall lower mortality rate, neurodegenerative mortality was 3 times greater than that in the general US population; for ALS and Alzheimer disease, the rate was 4 times higher. Similarly, a more recent study by Daneshvar and colleagues^30^ of all NFL players who began their professional careers between 1960 and 2019 found that age-, sex-, and race-adjusted mortality from ALS was approximately 4 times higher than that in the general US population. A recent study found that among former NFL players, those with higher estimated RHI exposure had an increased risk for overall mortality.^31^

Despite previous research focusing on the later-life effects of playing American football at the professional level, the long-term effects of college football participation remain largely unknown. Compared with professional football, football at the college level has been played by substantially more individuals.^32^ In 2020, there were 1536 players on NFL teams’ rosters.^33^ In contrast, the National Collegiate Athletic Association (NCAA) reported 73 137 active college football players.^34^ More than 800 000 student athletes have played football in US colleges since 1960, with more than 250 000 former college players currently older than 60 years (eMethods in the Supplement). From a public health perspective, elucidation of the long-term neurological and general health implications of playing football at the college level is needed.

Based on the limited studies to date, compared with the general population, individuals who played football through college, but not beyond, have increased risk for later-life cognitive impairment, depression and anxiety symptoms, and neurobehavioral dysregulation^4,35,36^ as well as structural brain alterations on neuroimaging.^37^ In 1 study of former college football players (mean age, 38 years),^38^ greater RHI exposure from football was significantly associated with smaller hippocampal volume. Former collegiate players may also be at increased risk for chronic traumatic encephalopathy.^16,17,39^ In a convenience sample of deceased college football players from a brain bank with acknowledged potential ascertainment bias,^16^ 48 of 53 (91%) were neuropathologically diagnosed with chronic traumatic encephalopathy. In a survey study of older former college athletes (mean age, 64 years),^35^ former football players reported significantly worse overall physical health, rated on a single 5-point Likert scale, than did other athletes who participated in noncontact sports and nonathletes. The goals of this study were to estimate the prevalence of self-reported health conditions, including cognitive and other neurological disorders, in older former college football players compared with men in the general population and to examine standardized mortality ratios (SMRs) and causes of death in former college football players.

## Methods

### University of Notre Dame Cohort

The target population for this cohort study was the 447 former University of Notre Dame (ND) football players who were listed as seniors on the varsity rosters during the football seasons from 1964 to 1980. The selection of this group of players was based on several factors: the age range at the time of study (59-75 years) was appropriate to investigate prevalence of age-related diseases^40^; former players from the earliest seasons represent the oldest group of living football players who likely played their entire football careers (including high school) wearing hard plastic helmets and face masks; there were only 2 coaches at ND during these 17 years, thus reducing variability in style of play and selection bias and potential unobserved confounders (eg, differences in playing style, rules, and equipment in different eras that would be directly associated with exposure and outcome); and grassroots support from a steering committee of former ND players increased the availability of accurate contact information of the cohort and increased the potential for a high response rate. History of playing professional football after college was determined by searching the Pro Football Reference website.^41^ Informed consent forms were presented online, and each participant was asked to select a response of “I agree” or “I do not wish to participate.” This was followed by six 2-choice questions to determine decisional capacity to provide consent. If all questions were not answered correctly (after 2 chances), the participant’s next of kin was asked to provide consent. This study was approved by the Boston University Medical Campus institutional review board. The study followed the Strengthening the Reporting of Observational Studies in Epidemiology (STROBE) reporting guideline for cohort studies.

### Health Survey

We developed an online survey (using REDCap^42^) that included 74 primary questions and additional possible follow-up questions. Items addressed general health conditions, including but not limited to diagnosis or treatment for cognitive, neurological, psychiatric, cardiovascular, orthopedic, and sleep conditions (eTable 1 in the Supplement). The survey also included the Satisfaction with Life Scale, a 5-item instrument measuring global judgments of satisfaction with one's life (scores range from 5 to 35, with higher scores indicating greater satisfaction).^43^

#### Former ND Football Player Health Survey Sample

We obtained contact information for 406 of the 447 former ND players or their next of kin if deceased through lists provided by the steering committee, searches of alumni newsletters, and queries of Whitepages.com. Participants were recruited by email, telephone, and/or mail solicitations. If a former player was deceased or did not meet predetermined decisional capacity standards, a family member was asked to complete the survey on the player’s behalf. Surveys were completed from December 2018 to May 2019.

#### Health and Retirement Study Sample

To compare the prevalence of medical conditions among the former ND football players with that among men in the general population, we used data from the Health and Retirement Study (HRS),^44^ a longitudinal study of a representative sample of US adults older than 50 years. The HRS is sponsored by the National Institute on Aging and conducted by the University of Michigan. University of Notre Dame survey questions were harmonized with HRS 2016 survey questions (eTable 1 in the Supplement).^44^ In both surveys, participants provided responses to questions about diagnoses and treatment provided by health care workers, with similar or identical wording used in the surveys. The ND survey included additional questions or more details for diagnostic categories for which there were no parallel HRS items.

### Standardized Mortality Ratios

The living status for all 447 former ND players in the cohort was confirmed using online sources (ie, obituaries, articles, and the alumni database) and/or the National Death Index. Demographic information, including dates of birth and death, were obtained through these sources. The National Death Index was then searched for the years 1979 to 2018 to collect underlying causes of death, reported as codes obtained from *International Statistical Classification of Diseases, Revision 7*; *International Statistical Classification of Diseases, Revision 8*; *International Classification of Diseases, Ninth Revision;* and *International Statistical Classification of Diseases and Related Health Problems, Tenth Revision*.

### Statistical Analysis

Of the 216 living ND respondents, 206 were matched to 3 HRS participants, and because of matching constraints, the remaining 10 ND respondents were matched to 2 HRS participants. The matching process used propensity scores based on age, sex, self-identified race, years of education (16 years or ≥17 years), employment status (full-time or part-time, retired, or disabled), and living situation (homeowner, renter, or other). Matching was conducted in R, version 3.6.1 (R Foundation for Statistical Computing)^45^ using the package MatchIT with a caliper of 0.1. For bivariate comparisons, we used a Wilcoxon rank sum test for continuous outcomes and a χ^2^ test of independence for categorical outcomes. All *P* values were adjusted for multiple comparisons with the false discovery rate using the Benjamini-Hochberg procedure.^46^ For all comparisons of health outcomes and prevalence rates, analyses were conducted in R (epiR package).^45^ A sensitivity analysis was conducted to examine the possible impact of nonresponse bias (ie, survey responders having different health outcomes than survey nonresponders). Inverse probability weights for each ND participant were calculated by logistic regression with years of senior football season at ND, number of varsity football seasons played at ND, senior season body mass index, position played, and professional vs nonprofessional status as variables and the response to the survey question as the outcome. Significance was set at 2-tailed *P* < .05.

Standardized mortality ratios were calculated based on age, sex, and race, using the Life Table Analysis System, LTAS.NET version 4.5.0^47^ developed by the National Institute for Occupational Safety and Health. Analyses used US male mortality rates (1960-2014) for 119 cause-of-death categories.^48^ To allow for direct comparisons with a study of former NFL players, mortality rates for 3 neurodegenerative causes of death were further evaluated using updated custom rate files used in previous studies.^29,49^

## Results

### Health Survey

Of the 406 individuals invited to participate in the survey, 234 (57.6%) responded, including 216 living former players and 18 individuals identified as next of kin of deceased players. Survey responders and nonresponders did not differ in year of their senior season, number of seasons played at ND, position played, and senior season body mass index. A greater proportion of nonresponders played professionally (eTable 2 in the Supplement). The 216 living ND players who completed the health survey (median age, 67 years; IQR, 63-70 years) were compared with 638 participants in the HRS (median age, 66 years; IQR, 63-70 years). Most ND players were White (200 [93%]), and 33 (15%) played professional football. Demographics of both samples are shown in Table 1, and a comparison of health conditions reported by participants in the samples is provided in Table 2. Compared with the HRS sample, former players reported a higher prevalence of cognitive impairment (10 [5%] vs 8 [1%]; *P* = .02), headaches (22 [10%] vs 22 [4%]; *P* = .001), CVD (70 [33%] vs 128 [20%]; *P* = .001), hypercholesterolemia (111 [52%] vs 182 [29%]; *P* = .001), and alcohol use (185 [86%] vs 489 [77%]; *P* = .02) and a lower prevalence of diabetes (24 [11%] vs 146 [23%]; *P* = .001). On the Satisfaction with Life Scale, ND players had a median score of 30 (IQR, 26-33), indicating being satisfied to highly satisfied with their life overall.^50^ Results of the sensitivity analysis for nonresponse bias indicated no meaningful changes to the health conditions results (eTable 3 in the Supplement). Additional analyses revealed no significant differences between former ND football players whose career stopped at the college level (n = 183) and those who played professionally (n = 33) in demographics (eTable 4 in the Supplement) or health outcomes (eTable 5 in the Supplement). There were no significant differences in health conditions between responders who played different positions (eTable 6 in the Supplement).

**Table 1.** Demographics of the University of Notre Dame and HRS Samples^a^

Characteristic	Notre Dame (n = 216)^b^	HRS (n = 638)	*P* value	FDR-adjusted *P* value^c^
Age, median (IQR), y	67 (63-70)	66 (63-70)	.52	.86
Race				
African American or Black	12 (6)	NA^d^	NA^d^	NA^d^
White^e^	200 (93)	585 (92)	.67	.86
Multiracial	1 (1)	NA^d^	NA^d^	NA^d^
Declined to answer	3 (1)	NA^d^	NA^d^	NA^d^
Education, y				
16	89 (41)	318 (50)	.03	.17
≥17	127 (59)	320 (50)		
Highest level of education				
Bachelor’s degree	113 (52)	NA	NA	NA
Master’s degree	51 (24)	NA	NA	NA
Doctoral or professional degree	52 (24)	NA	NA	NA
Married or domestic partnership	180 (84)	532 (83)	.89	.86
Employment status				
Full-time or part-time	123 (57)	370 (58)	.86	.86
Retired	89 (41)	259 (41)		
Disabled	4 (2)	9 (1)		
Living situation				
Homeowner	200 (93)	602 (94)	.57	.89
Renter	10 (4.6)	20 (3.1)		
Other	6 (2.8)	16 (2.5)		
Senior year BMI, median (IQR)	27.7 (25.8-29.5)	NA	NA	NA
Position played^f^				
Lineman or linebacker	115 (53)	NA	NA	NA
Back or receiver	83 (38)	NA	NA	NA
Quarterback	9 (4)	NA	NA	NA
Kicker	9 (4)	NA	NA	NA
Professional football experience	33 (15)	NA	NA	NA

Abbreviations: BMI, body mass index (calculated as weight in kilograms divided by height in meters squared); FDR, false discovery rate; HRS, Health and Retirement Study; NA, not available.

^a^ The χ^2^ test of independence and Fisher exact test were performed. Data are presented as number (percentage) of individuals unless otherwise indicated.

^b^ Data reported for living Notre Dame players who completed the survey.

^c^ False discovery rate correction for multiple testing.

^d^ Because the number of participants was small, propensity score matching could not include the specific data for the group.

^e^ One individual in the Notre Dame sample identified as Hispanic White.

^f^ Lineman or linebacker and back or receiver groups include both offensive and defensive positions.

**Table 2.** Health Outcomes in the University of Notre Dame and HRS Samples^a^

Health outcome	Notre Dame (n = 216)^b^	HRS (n = 638)	*P* value	FDR-adjusted *P* value^c^
Neurological disorders				
Cognitive impairment diagnosis	10 (5)	8 (1)	.01	.02
Parkinson disease	1 (1)			
Recurrent headaches	22 (10)	22 (4)	<.001	.001
Vascular conditions				
Cardiovascular disease	70 (33)	128 (20)	<.001	.001
Heart attack history	11 (5)	NA	NA	NA
Abnormal heart rhythm	44 (21)	NA	NA	NA
Artery blockage	33 (15)	NA	NA	NA
Valve problem	9 (4)	NA	NA	NA
Heart failure	7 (3)	NA	NA	NA
Heart surgery	30 (14)	NA	NA	NA
Cardiovascular risk factors	150 (70)	429 (68)	.49	.60
High blood pressure	102 (48)	340 (54)	.13	.24
High cholesterol level	111 (52)	182 (29)	.001	<.001
Diabetes	24 (11)	146 (23)	<.001	.001
Stroke	6 (3)	31 (5)	.27	.36
Psychiatric diagnosis				
Any	35 (17)	112 (18)	.87	.92
Depression	30 (14)	94 (15)	.92	.92
Anxiety	17 (8)	NA	NA	NA
Bipolar disorder	1 (1)	NA	NA	NA
Psychosis or schizophrenia	0	NA	NA	NA
Neurodevelopmental diagnosis				
ADHD	6 (3)	NA	NA	NA
Dyslexia	5 (2)	NA	NA	NA
Sleep disorders				
Sleep apnea	58 (27)	124 (19)	.03	.06
Restless leg syndrome	2 (1)	NA	NA	NA
REM sleep behavior disorder	5 (2)	NA	NA	NA
Orthopedic				
Joint replacement	73 (34)	NA	NA	NA
Back surgery	24 (11)	NA	NA	NA
Other orthopedic surgery	127 (60)	NA	NA	NA
Other medical				
Cancer^d^	35 (17)	83 (13)	.16	.26
Lung condition	17 (8)	27 (4)	.06	.11
Liver disease	8 (4)	NA	NA	NA
Kidney disease	12 (6)	NA	NA	NA
Gastrointestinal disorder	28 (13)	NA	NA	NA
Eye disorder	47 (22)	NA	NA	NA
Thyroid condition	27 (13)	NA	NA	NA
Low testosterone	15 (7)	NA	NA	NA
Alcohol use				
Any	185 (86)	489 (77)	.01	.02
Frequency among users				
≤1 time/mo	25 (14)	82 (17)	.57	.65
2-4 times/mo	45 (24)	114 (23)		
2-3 times/wk	55 (30)	124 (25)		
≥4 times/wk	60 (32)	167 (34)		
Drinks, No.				
1 or 2	137 (74)	324 (80)	.24	.35
3 or 4	40 (22)	65 (16)		
≥5	7 (4)	14 (4)		
Drug use				
Illicit drug use	104 (49)	NA	NA	NA
Prescription drug abuse	11 (5)	NA	NA	NA
Performance-enhancing drugs	10 (5)	NA	NA	NA
Satisfaction with life score, median (IQR)^e^	30 (26-33)	NA	NA	NA

Abbreviations: ADHD, attention-deficit/hyperactivity disorder; FDR, false discovery rate; HRS, Health and Retirement Study; NA, not available; REM, rapid eye movement.

^a^ The χ^2^ test of independence and Fisher exact test were performed. Data are presented as number (percentage) of individuals unless otherwise indicated. Specific wording of questions is provided in eTable 1 in the Supplement.

^b^ Data reported for living Notre Dame players who completed the survey.

^c^ False discovery rate correction for multiple testing.

^d^ Does not include nonmelanoma skin cancer.

^e^ Based on the Satisfaction with Life Scale (scores range from 5 to 35, with higher scores indicating greater satisfaction).^43^

### Standardized Mortality Ratios

Of the 447 former ND players, 76 (17%) were reported to be deceased as of May 5, 2020. Standardized mortality ratios of all-cause and specific causes of death among ND players compared with those among men in the general US population are reported in Table 3. Only underlying-cause SMRs are presented. All-cause mortality (SMR, 0.54; 95% CI, 0.42-0.67) and mortality from heart (SMR, 0.64; 95% CI, 0.39-0.99), circulatory (SMR, 0.23; 95% CI, 0.03-0.83), respiratory (SMR, 0.13; 95% CI, 0.00-0.70), and digestive system (SMR, 0.13; 95% CI, 0.00-0.74) disorders; lung cancer (SMR, 0.26; 95% CI, 0.05-0.77); and violence (SMR, 0.10; 95% CI, 0.00-0.58) were significantly lower in the ND cohort than in the general population. Mortality from brain and other nervous system cancers was significantly higher in the ND cohort (SMR, 3.82; 95% CI, 1.04-9.77). Although point estimates for all neurodegenerative causes (SMR, 1.42; 95% CI, 0.29-4.18) and Parkinson disease (SMR, 2.07; 95% CI, 0.05-11.55) were higher in the ND cohort, the difference did not reach statistical significance. A total of 2 former ND players (0.5%) in the cohort died of ALS, with an SMR of 2.93 (95% CI, 0.36-10.59); mortality was not significantly higher in the ND cohort. Multiple-cause data were also assessed and did not meaningfully change the results.

**Table 3.** Standardized Mortality Ratios in the Overall Cohort of 447 Former University of Notre Dame Football Players^a^

Cause of death	Players, No.	SMR (95% CI)
All causes	76	0.54 (0.42-0.67)^b^
Cancer		
All	24	0.68 (0.43-1.01)
Buccal and pharynx	2	1.91 (0.23-6.91)
Digestive and peritoneum	7	0.65 (0.26-1.34)
Respiratory	3	0.26 (0.05-0.77)^b^
Male genital organs	1	0.51 (0.01-2.84)
Urinary	1	0.58 (0.01-3.24)
Melanoma	2	3.48 (0.42-12.58)
Mesothelioma	1	7.66 (0.19-42.70)
Brain and other nervous system	4	3.82 (1.04-9.77)^b^
Other and unspecified	0	0.00 (0.00-1.40)
Lymphatic and hematopoietic	3	0.89 (0.18-2.60)
Diseases of blood and blood-forming organs	0	0.00 (0.00-5.88)
Diabetes	2	0.42 (0.05-1.53)
Mental, psychoneurotic, and personality disorders	1	0.35 (0.01-1.93)
Diseases of the nervous system and sense organs		
All	4	1.46 (0.40-3.74)
Neurodegenerative causes^c^	3	1.42 (0.29-4.18)
Alzheimer disease	0	0.00 (0.00-3.94)
Amyotrophic lateral sclerosis	2	2.93 (0.36-10.59)
Parkinson disease	1	2.07 (0.05-11.55)
Other diseases of the nervous system and sense organs	1	0.64 (0.04-8.70)
Diseases of the heart	20	0.64 (0.39-0.99)^b^
Other diseases of the circulatory system	2	0.23 (0.03-0.83)^b^
Diseases of the respiratory system	1	0.13 (0.00-0.70)^b^
Diseases of the digestive system	1	0.13 (0.00-0.74)^b^
Diseases of musculoskeletal and connective tissue	0	0.00 (0.00-8.43)
Diseases of the genitourinary system	0	0.00 (0.00-1.40)
Symptoms and ill-defined conditions	0	0.00 (0.00-1.69)
Transportation injuries	2	0.30 (0.04-1.08)
Other injuries		
All	2	0.32 (0.04-1.16)
Drowning	1	1.47 (0.04-8.17)
Accidental poisoning	1	0.43 (0.01-2.38)
Violence		
All	1	0.10 (0.00-0.58)^b^
Intentional self-harm	1	0.24 (0.01-1.31)
Assault and homicide	0	0.00 (0.00-0.68)^b^
Other and unspecified causes	3	0.51 (0.10-1.49)
Unknown	13	NA^d^

Abbreviations: NA, not applicable; SMR, standardized mortality ratio.

^a^ Standardized mortality ratios were calculated based on age, sex, and race with the Life Table Analysis System^47^ using US male mortality rates (1960-2014) for 119 cause-of-death categories. Only underlying-cause SMRs are presented. Multiple-cause data were also assessed and did not meaningfully change the results.

^b^ Significant difference (*P* < .05).

^c^ Standardized mortality ratios for 3 neurodegenerative causes of death assessed with updated custom rate files used in previous studies.^29,48^

^d^ The Life Table Analysis System^47^ software does not calculate SMRs for unknown causes of death.

## Discussion

To our knowledge, this study is the first to compare the prevalence of later-life health conditions in a well-defined cohort of older, former college football players with that in a matched group from the general population. To our knowledge, it is also the first to compare the mortality rates between former college football players and the general US male population. Former college players had a significantly higher prevalence of self-reported cognitive impairment diagnoses, recurrent headaches, and CVD compared with a matched sample from the HRS. Consistent with a previous report on former NFL players,^51^ former college players reported a significantly greater prevalence of hypercholesterolemia but a lower prevalence of diabetes. Also consistent with studies of former NFL players,^20^ the former college players reported a higher prevalence of sleep apnea, although the result was not statistically significant after false discovery rate adjustment for multiple comparisons. In contrast to previous reports of mood and behavioral impairments in both former professional and college football players,^4,5,6,7,36^ in the present study, there were no significant group differences in psychiatric diagnoses between the ND group and the HRS comparison group. This difference in findings may be attributable to the specific wording of the survey questions, which asked about actual diagnoses, whereas previous studies relied on scores on mood and behavior measures. There were no significant differences in the prevalence of cancer or lung conditions between the ND and HRS groups.

All-cause mortality in the ND sample was significantly lower than that in the general US population. The former college players had significantly lower mortality from CVD despite the health survey results indicating higher prevalence of self-reported CVD compared with the general population. This discrepancy has also been observed in former NFL players^23,27^ and requires additional investigation. Also similar to reports of former NFL players,^27,28^ the former college players had significantly lower mortality from diseases of the circulatory, respiratory, and digestive systems and from violence, including intentional self-harm. There was no difference in overall mortality from cancer, although mortality from lung cancer was significantly lower among the former players than in the general US population. Brain and other nervous system cancer mortality rates were significantly higher among the former college football players. In addition, mortality from all neurodegenerative causes and specific neurodegenerative diseases was higher compared with that in the general US population, but the difference did not reach statistical significance.

Several of the lower SMRs in the former college player cohort are consistent with results in studies of former NFL players^25,26,27,28,29,52^ and may be associated with the “healthy worker effect,” including regular exercise, physical activity, and lower cigarette smoking rates among male college athletes.^53^ Results are also similar to those of recent studies of former professional soccer players in Scotland,^54,55^ which found lower overall mortality and mortality from ischemic heart disease and lung cancer compared with the general population. However, in those studies of former Scottish soccer players, mortality from neurodegenerative disease (including Alzheimer disease, ALS, and Parkinson disease) was higher than that in the general population.^54,55^ This finding may have been associated with history of exposure to RHI (ie, from purposeful heading of the ball).^55,56^ Similarly, elevated neurodegenerative disease mortality in former NFL players has been hypothesized to be associated with RHI exposure incurred through routine play of football (ie, head impacts resulting in the common subconcussive trauma and not just symptomatic concussions or more severe brain injuries) since the adoption of hard plastic helmets and face masks in the late 1950s.^29,57^

In the current study, 2 former ND players (0.5%) in the cohort died of ALS, with an SMR of 2.93 (95% CI, 0.36-10.59). Although the SMR did not reach significance, the findings are consistent with those of previous studies, and we believe they warrant discussion. The prevalence of ALS in the general US population is 5 per 100 000 population.^58^ An association between RHI and ALS has been suggested based on findings of comorbid chronic traumatic encephalopathy and ALS pathology in former football players and others with RHI exposure.^19,59^ Earlier studies reported that former NFL players had unusually high prevalence of ALS compared with the general population (eg, 206 per 100 000 former NFL players^18^) and that the SMR for ALS in former NFL players was 4.31 (95% CI, 1.73-8.87).^29^ A recent cohort study^30^ of 19 423 current and former NFL players found significantly higher incidence (standardized incidence ratio, 3.59; 95% CI, 2.58-4.93) and mortality (SMR, 3.94; 95% CI, 2.62-5.69) of ALS among NFL players compared with the US male population after adjustment for age, sex, and race. In addition, the studies of former professional Scottish soccer players found a 3.5 greater risk of mortality from ALS compared with that in the general population,^54,55^ and former Italian professional soccer players have been found to have a 1.5 to 6.5 greater risk of ALS compared with the general population^60,61^ and an 11.5 to 18.2 increased ALS mortality rate.^62,63^ A systematic review of studies examining risk for ALS among participants in contact sports concluded that sports with exposure to repetitive concussive head and neck trauma were associated with significantly increased risk for ALS and that there was a combined 8.5-fold increased risk associated with professional American football and soccer.^64^ Future research is needed to examine further the possible association between playing college football and subsequent increased risk of ALS.

It is unclear why brain and other nervous system cancer mortality was higher in the ND cohort than in the general US male population. To our knowledge, no studies have found a direct association between repetitive mild brain injuries and brain cancer. The association between a single traumatic brain injury and subsequent brain cancer has been reported,^65^ although this finding has been equivocal.^66^ Future studies are required to examine this possible association further.

### Strengths and Limitations

This study has strengths. These included sampling from a well-characterized population of older former college football athletes who played during a defined period, a comparison of health outcomes between living former college players and a matched sample from the general population, and a comparison of all-cause mortality and cause-specific mortality between former college players and the general US male population.

This study also has limitations. The relative homogeneity of the cohort decreases the generalizability of the findings. More than 80% of the ND student body is Catholic.^67^ This may, in part, be associated with the relatively high levels of reported satisfaction with life in the sample; previous studies reported an association of religious and spiritual identity with overall satisfaction with life.^68^ The ND cohort was 93% White, which further limits generalizability of these results to former players of the same era who attended schools with greater racial and ethnic diversity at the time and precluded our ability to examine the association of race, structural racism, and other dimensions of racism and related disparities in social determinants of health with health outcomes.^69,70^

A total of 15% of the living ND respondents went on to play football professionally. We did not find any significant health outcome differences between survey respondents who played professionally and those who did not after false discovery rate adjustment for multiple comparisons. eTable 7 in the Supplement gives the SMR results for the members of the ND cohort who did not play professionally and a comparison with previous reports of mortality among former NFL players by Lincoln et al^27^ and Lehman et al.^29^ The SMR results of the former college players are similar to those of the former professionals overall. However, factors related to playing professionally (eg, additional years of RHI exposure and other injuries, additional years of exercise and fitness) may be associated with negative or positive later-life health outcomes and mortality. Future studies should include former players from schools and divisions with less competitive football programs and fewer students who go on to play professionally. The results of this study may also not be representative of former athletes who played more recently. Changes in football equipment, rules, and player habitus and speed^71^ have changed over the past 5 to 6 decades. Future studies should include players from different eras of play to examine the impact of these changes.

The completion rate for our health survey (57.6%) was relatively high compared with that of other surveys of former college football players (eg, 23.4%)^72^ or NFL players (eg, 25.6%).^6^ Nonetheless, the absence of responses from 42.4% of eligible persons means that nonresponse bias warrants consideration. Although our sensitivity analysis findings did not support the presence of large bias from nonresponse (eTable 3 in the Supplement), the analysis was based solely on the characteristics of ND players for whom we had data, and we did not evaluate the characteristics of persons who opted not to enroll in HRS and complete its corresponding health survey. It remains possible that nonresponding ND players were disproportionately affected by high symptom burdens that precluded their ability to participate. If this were the case, outcome risks among the former players would tend to be underestimated, resulting in underestimated risks and exaggerated benefits of playing. In addition, the use of self-reported medical history may have resulted in inaccurate reporting.^73,74^ Another important limitation to this study is the small total size of the population of interest (n = 447); our analyses may be underpowered for cause-specific events.

## Conclusions

More than 800 000 individuals have played college football in the US since 1960, with 73 000 or more active college players each year since 2016.^34^ Despite important gains in helmet and other protective technology as well as rule changes meant to improve the overall safety of the game, US football remains a violent collision sport. Compared with other college sports, football has the highest rate of injuries, with 39.9 per 1000 competition athlete exposures.^75^ The bigger helmets do not prevent the brain from rapid acceleration-deceleration movements in the skull, and college football players continue to experience a mean of approximately 600 head impacts per season.^76^ Regarding rule changes, a recent reduction in the number of allowed preseason on-field practices was associated with an increase in total preseason head impact burden in some teams owing to longer and more intense practices.^77^ In this cohort study, playing college football was associated with both positive and negative long-term health outcomes. With the increasing number of living former college football players, additional research is needed to expand on our findings and to provide all stakeholders with accurate, unbiased data and guidance to maximize factors that improve later-life health outcomes and eliminate or reduce factors that may increase risk for later-life morbidity and mortality.

## Notes

Supplement. **eMethods.** Estimation of the Number of Former College Football Players Who Played in American Colleges between 1960 and 2020 and of Former Players who are Ages 60 to 82 **eTable 1.** University of Notre Dame (ND) Sample Survey Questionnaire and Health and Retirement Study (HRS) 2016 Survey: Variable Matching **eTable 2.** Comparison of Former University of Notre Dame (ND) Players Who Completed the Survey and Those Who Did Not Complete the Survey **eTable 3.** Non-Responder Weighted Health Outcomes: University of Notre Dame (ND) Players Compared with Health and Retirement Study (HRS) Sample **eTable 4.** Demographics of Former University of Notre Dame (ND) Players Who Only Played Through College Compared with Those Who Went on to Play Professional Football **eTable 5.** Health Outcomes: Former University of Notre Dame (ND) Players Who Only Played Through College Compared with Those Who Went on to Play Professional Football **eTable 6.** Health Outcomes: Former University of Notre Dame (ND) Players by Position **eTable 7.** Standardized Mortality Ratios for Former University of Notre Dame (ND) Players Who Only Played Through College Compared with Other Studies of Former National Football League (NFL) Players
eReferences.
