# Supplementary material for: Association of Playing College American Football With Long-term Health Outcomes and Mortality
Source: JAMA Netw Open. 2022 Apr 20;5(4):e228775. doi: 10.1001/jamanetworkopen.2022.8775 (PMC9021915; doi:10.1001/jamanetworkopen.2022.8775)
Supplement: Supplement. — eMethods. Estimation of the Number of Former College Football Players Who Played in American Colleges between 1960 and 2020 and of Former Players who are Ages 60 to 82 eTable 1. University of Notre Dame (ND) Sample Survey Questionnaire and Health and Retirement Study (HRS) 2016 Survey: Variable Matching eTable 2. Comparison of Former University of Notre Dame (ND) Players Who Completed the Survey and Those Who Did Not Complete the Survey eTable 3. Non-Responder Weighted Health Outcomes: University of Notre Dame (ND) Players Compared with Health and Retirement Study (HRS) Sample eTable 4. Demographics of Former University of Notre Dame (ND) Players Who Only Played Through College Compared with Those Who Went on to Play Professional Football eTable 5. Health Outcomes: Former University of Notre Dame (ND) Players Who Only Played Through College Compared with Those Who Went on to Play Professional Football eTable 6. Health Outcomes: Former University of Notre Dame (ND) Players by Position eTable 7. Standardized Mortality Ratios for Former University of Notre Dame (ND) Players Who Only Played Through College Compared with Other Studies of Former National Football League (NFL) Players eReferences. [file jamanetwopen-e228775-s001.pdf]

Supplementary Online Content

Phelps A, Alosco ML, Baucom Z, et al. Association of playing college American football with long-term health outcomes and mortality. *JAMA Netw Open*. 2022;5(4):e228775. doi:10.1001/jamanetworkopen.2022.8775

eMethods. Estimation of the Number of Former College Football Players Who Played in American Colleges between 1960 and 2020 and of Former Players who are Ages 60 to 82
eTable 1. University of Notre Dame (ND) Sample Survey Questionnaire and Health and Retirement Study (HRS) 2016 Survey: Variable Matching
eTable 2. Comparison of Former University of Notre Dame (ND) Players Who Completed the Survey and Those Who Did Not Complete the Survey
eTable 3. Non-Responder Weighted Health Outcomes: University of Notre Dame (ND) Players Compared with Health and Retirement Study (HRS) Sample
eTable 4. Demographics of Former University of Notre Dame (ND) Players Who Only Played Through College Compared with Those Who Went on to Play Professional Football
eTable 5. Health Outcomes: Former University of Notre Dame (ND) Players Who Only Played Through College Compared with Those Who Went on to Play Professional Football
eTable 6. Health Outcomes: Former University of Notre Dame (ND) Players by Position
eTable 7. Standardized Mortality Ratios for Former University of Notre Dame (ND) Players Who Only Played Through College Compared with Other Studies of Former National Football League (NFL) Players
eReferences.

This supplementary material has been provided by the authors to give readers additional information about their work.

© 2022 Phelps A et al. *JAMA Network Open*.

eMethods. Estimation of the Number of Former College Football Players Who Played in American Colleges between 1960 and 2020 and of Former Players who are Ages 60 to 82.

The NCAA Sports Sponsorship and Participation Rates report and database[1] have reported annual counts of the Number of Teams, Total Athletes, and Average Squad Size for collegiate American football every five years between 1957 and 1977 and later annually beginning in 1982. These three categories are not *explicitly* related, but it can be closely estimated that Number of Teams multiplied by Average Squad Size would result in Total Athletes.

Total Athletes = Number of Teams x Average Squad Size

To estimate the Total Athletes over the age of 60, this formula was applied to the years 1960 to 1981 when annual NCAA data was not available. Assuming senior roster players were 22 years old, we set a cutoff at the year 1960 to include all individuals less than or equal to 82 years old as of 2020. The Number of Teams was based on those in major, minor, and independent American football conferences across randomly sampled years which was then extrapolated based on averages. Similarly, Average Squad Size was estimated by randomly sampling rosters from various teams in various years and extrapolating. At this point, Total Athletes could be estimated.

It is important to note that early college football included separate freshmen and varsity teams until the NCAA allowed freshmen to join the varsity rosters in 1972. Because we do not have access to freshmen team rosters, we calculated the total number players assuming individuals played three years of varsity football prior to 1972 and four years including and after 1972. It is important to note that we are potentially overestimating by assuming one third of the roster were seniors in the early college football years, rather than one fourth due to the inclusion of freshman. However, the average number of years on the varsity roster in our study sample was three, which further justifies our calculations.

Total Athletes Between 1960 and 2020 (Seasons 1960-2020) =
(1960 to 1971: Number of Teams x Average Squad Size x 1/3) + (1972 to 2020: Number of Teams x Average Squad Size x 1/4)
= 814,122

Total Athletes Ages 60 to 82 as of 2020 (Seasons 1960-1982) =
(1960 to 1971: Number of Teams x Average Squad Size x 1/3) + (1972 to 1982: Number of Teams x Average Squad Size x 1/4) =
= 251,309

© 2022 Phelps A et al. *JAMA Network Open*.

eTable 1. University of Notre Dame (ND) Sample Survey Questionnaire and Health and Retirement Study (HRS) 2016 Survey: Variable Matching

Characteristic (Matched Variable)	ND Variable	ND Question	HRS Variable	HRS Question
**Age**	age	How old are you currently?	PA019	Current Age Calculation
**Race**	raceusacat___1, raceusacat___2, raceusacat___3, raceusacat___4	What do you consider your current race?	PB089M1M PB089M2M PB089M3M	What race do you consider yourself to be?
**Highest level of Education**	edulevel	What is the highest grade or level of school you have completed or the highest degree obtained?	PB017M	What is the highest degree you have earned?
**Married/Domestic partnership**	marital	What is your current marital or partner status?	PB063	Marital Status Assigned
**Employment status**	employ	What is your current employment status?	PJ005M1	Are you working now, temporarily laid off, unemployed and looking for work, disabled and unable to work, retired, a homemaker, or what?
**Living situation**	home	What is your current living situation?	PH004	Do you [and your] [husband/wife/partner] own your home, rent it, or what?
**Cognitive impairment**	dxd	Have you ever been diagnosed with, or treated for dementia, Alzheimer's disease, CTE, mild cognitive impairment, or something similar by a doctor or other healthcare provider?	PC273	Has a doctor ever told you that you have dementia, senility or any other serious memory impairment?
**Cardiovascular disease**	heartdx	*If "yes" to any of the following:*<br>Have you ever had a heart attack or myocardial infarction (MI)?<br>Have you ever been diagnosed with coronary artery disease and/or blocked blood vessels to the heart?<br>Have you ever been diagnosed with angina?<br>Have you ever been diagnosed with congestive heart failure (CHF)?	PC036	Has a doctor ever told you that you have had a heart attack, coronary heart disease, angina, congestive heart failure, or other heart problems?

© 2022 Phelps A et al. *JAMA Network Open*.

		Have you ever had or been in cardiac arrest (separate from heart attack above)?<br>Have you ever been formally diagnosed with atrial fibrillation (AFib)?<br>Have you ever been diagnosed with cardiomyopathy?<br>Have you ever been diagnosed with valvular heart disease, or bad heart valve?<br>Have you ever been diagnosed with congenital heart disease (present at birth)?		
**Cardiovascular risk factors**	hbp, hchol, diab	*If "yes" to any of the following:*<br>Have you ever been formally diagnosed or treated with high blood pressure or hypertension?<br>Have you ever been formally diagnosed or treated for high cholesterol?<br>Have you ever been formally diagnosed or treated for diabetes?	PC005, PC283, PC010	*If "yes" to any of the following:*<br>Has a doctor ever told you that you have high blood pressure or hypertension?<br>Have you ever been told by a doctor or other health professional that your blood cholesterol level was high?<br>Has a doctor ever told you that you have diabetes or high blood sugar?
**High blood pressure**	hbp	Have you ever been formally diagnosed or treated with high blood pressure or hypertension?	PC005	Has a doctor ever told you that you have high blood pressure or hypertension?
**High cholesterol**	hchol	Have you ever been formally diagnosed or treated for high cholesterol?	PC283	Have you ever been told by a doctor or other health professional that your blood cholesterol level was high?
**Diabetes**	diab	Have you ever been formally diagnosed or treated for diabetes?	PC010	Has a doctor ever told you that you have diabetes or high blood sugar?
**Stroke**	strktiatyp___1	*Have you ever had a stroke or transient ischemic attack (TIA)? If yes:*<br>What event(s) did you have?<br>Please check [+] all that apply.<br>(choice=Stroke(s))	PC053	Has a doctor ever told you that you have had a stroke?
**Psychiatric diagnosis**	psychdx	*If "yes" to any of the following:*<br>Have you ever been formally diagnosed or treated for depression?<br>Have you ever been formally diagnosed or treated with anxiety?	PC065	Have you ever had or has a doctor ever told you that you have any emotional, nervous, or psychiatric problems?

© 2022 Phelps A et al. *JAMA Network Open*.

		Have you ever been formally diagnosed or treated with Bipolar Disorder or Manic Depression?<br>Have you ever been formally diagnosed or treated with Psychosis or Schizophrenia?<br>Have you ever been formally diagnosed or treated with Post Traumatic Stress Disorder, also known as PTSD?<br>Have you ever been diagnosed or treated for any other psychiatric, behavioral, or mood disorders?		
**Depression**	dpr	Have you ever been formally diagnosed with or treated for depression?	PC271	Has a doctor ever told you that you have had problems with depression?
**Sleep apnea**	slapn	Have you ever been diagnosed with sleep apnea?	PC292	What was the sleep disorder? (choice=1.Sleep Apnea)
**Cancer**	cancer	Have you ever been diagnosed with cancer?	PC018	Has a doctor ever told you that you have cancer or a malignant tumor, excluding minor skin cancer?
**Lung condition**	lung	Have you ever been diagnosed with lung or pulmonary condition or disease (other than cancer)?	PC030	Has a doctor ever told you that you have chronic lung disease such as chronic bronchitis or emphysema?
**Alcohol use**	alcfreq	How often do you have a drink containing alcohol?	PC128	Do you ever drink any alcoholic beverages such as beer, wine, or liquor?
**Alcohol frequency**	alcfreq	How often do you have a drink containing alcohol?	PC129	In the last three months, on average, how many days per week have you had any alcohol to drink? (For example, beer, wine, or any drink containing liquor.)
**Alcohol number of drinks**	alcnum	How many drinks containing alcohol do you have on a typical day when you are drinking?	PC130	In the last three months, on the days you drink, about how many drinks do you have?

HRS = Health and Retirement Study; ND = University of Notre Dame

© 2022 Phelps A et al. *JAMA Network Open*.

eTable 2. Comparison of Former University of Notre Dame (ND) Players Who Completed the Survey and Those Who Did Not Complete the Survey[a]

	Completers N=234	Non-Completers N=213	*P* value	FDR Adjusted[b]
**Living Status, n (%)**			**<0.01**	**<0.01**
Alive	216 (92)	155 (73)		
Deceased	18 (8)	58 (27)		
**Senior Season, n (%)**			0.06	0.12
1964	7 (3)	17 (8)		
1965	6 (3)	14 (7)		
1966	15 (6)	13 (6)		
1967	15 (6)	8 (4)		
1968	14 (6)	12 (6)		
1969	13 (6)	15 (7)		
1970	16 (7)	11 (5)		
1971	16 (7)	20 (9)		
1972	16 (7)	13 (6)		
1973	14 (6)	20 (9)		
1974	18 (8)	9 (4)		
1975	18 (8)	8 (4)		
1976	20 (9)	12 (6)		
1977	14 (6)	7 (3)		
1978	7 (3)	14 (7)		
1979	14 (6)	11 (5)		
1980	11 (5)	9 (4)		
**Number of Varsity Seasons at ND, n (%)**			0.52	0.62
1	10 (4)	9 (4)		
2	20 (9)	19 (9)		
3	188 (80)	162 (76)		
4	16 (7)	23 (11)		
**Senior Year BMI, median (IQR)**	27.8 (26.0, 29.5)	27.8 (25.8, 29.9)	0.33	0.50
**Position Played, n (%)**			0.80	0.80
Lineman/Linebacker	126 (54)	115 (54)		
Back/Receiver	89 (38)	82 (38)		

© 2022 Phelps A et al. *JAMA Network Open*.

	Quarterback	10 (4)	11 (5)		
	Kicker	9 (4)	5 (2)		
**Professional Football Experience, n (%)**		38 (16)	62 (29)	**<0.01**	**<0.01**

[a]Data reported for living and deceased ND players regardless of survey completion status.
[b]FDR = False discovery rate correction for multiple testing
BMI = Body mass index; FDR = False discovery rate; IQR = Interquartile range; ND = University of Notre Dame

© 2022 Phelps A et al. *JAMA Network Open*.

eTable 3. Non-Responder Weighted Health Outcomes: University of Notre Dame (ND) Players Compared with Health and Retirement Study (HRS) Sample[a]

Health Outcome Variable	ND n=216[b]	HRS n=638	*P* value	FDR Adjusted[c]
**Neurological disorders, n (%)**				
**Cognitive impairment diagnosis**	11 (5)	8 (1)	**<0.01**	**<0.01**
Parkinson's disease[d]	1 (1)			
**Recurrent headaches**	22 (10)	22 (4)	**<0.01**	**<0.01**
**Vascular conditions, n (%)**				
**Cardiovascular disease**	70 (33)	128 (20)	**<0.01**	**<0.01**
Heart attack history[d]	11 (5)			
Abnormal heart rhythm[d]	44 (21)			
Artery blockage[d]	33 (15)			
Valve problem[d]	9 (4)			
Heart failure[d]	7 (3)			
Heart surgery[d]	30 (14)			
Cardiovascular risk factors	150 (70)	429 (68)	0.45	0.55
High blood pressure	101 (47)	340 (54)	0.09	0.15
**High cholesterol**	112 (53)	182 (29)	**<0.01**	**<0.01**
**Diabetes**	24 (11)	146 (23)	**<0.01**	**<0.01**
Stroke	6 (3)	31 (5)	0.25	0.35
**Psychiatric diagnosis, n (%)**	37 (18)	112 (18)	0.96	0.96
Depression	32 (15)	94 (15)	0.85	0.90
Anxiety[d]	17 (8)			
Bipolar disorder[d]	1 (1)			
Psychosis/Schizophrenia[d]	0 (0)			
**Neurodevelopmental diagnosis, n (%)**				
ADHD[d]	6 (3)			
Dyslexia[d]	5 (2)			
**Sleep diagnosis, n (%)**				
**Sleep apnea**	58 (27)	124 (19)	**0.02**	**0.05**

© 2022 Phelps A et al. *JAMA Network Open*.

Restless leg syndrome[d]	2 (1)			
REM sleep behavior disorder[d]	5 (2)			
**Orthopedic, n (%)**				
Joint replacement[d]	73 (34)			
Back surgery[d]	24 (11)			
Other orthopedic surgery[d]	127 (60)			
**Other medical, n (%)**				
Cancer[e]	33 (16)	83 (13)	0.22	0.35
Lung condition	17 (8)	27 (4)	**0.04**	0.07
Liver disease[d]	8 (4)			
Kidney disease[d]	12 (6)			
Gastrointestinal disorder[d]	28 (13)			
Eye disorder[d]	47 (22)			
Thyroid condition[d]	27 (13)			
Low testosterone[d]	15 (7)			
**Alcohol use, n (%)**	184 (85)	489 (77)	**0.01**	**0.03**
Frequency among users			0.60	0.69
Monthly or less	25 (14)	82 (17)		
2-4 times a month	45 (24)	114 (23)		
2-3 times a week	55 (30)	124 (25)		
4+ times a week	60 (32%	167 (34)		
Number of drinks when drinking			0.26	0.35
1 or 2	137 (74)	324 (80)		
3 or 4	40 (22)	65 (16)		
5+	7 (4)	14 (4)		
**Drug use, n (%)**				
Illicit drug use[d]	104 (49)			
Prescription drug abuse[d]	11 (5)			
Performance enhancing drugs[d]	10 (5%			

[a]Specific wording of questions are provided in eTable 1.
[b]Data reported for living ND players who completed the survey.

© 2022 Phelps A et al. *JAMA Network Open*.

[c]FDR = False discovery rate correction for multiple testing
[d]HRS data not available.
[e]Does not include non-melanoma skin cancer.
Note: This sensitivity analysis was conducted to examine the possible impact of a nonresponse bias (i.e., survey responders having different health outcomes than survey nonresponders). Inverse probability weights for each ND subject were calculated by running a logistic regression, with years of senior football season at ND, number of varsity football seasons played at ND, senior season BMI, position played, and professional versus non-professional as predictors, and the response to the survey question as the outcome variable.
Statistics presented: n (%); median (IQR), Statistical tests performed: chi-square test of independence; Fisher’s exact test.
ADHD = Attention deficit hyperactivity disorder; BMI = body mass index; FDR = False discovery rate

© 2022 Phelps A et al. *JAMA Network Open*.

eTable 4. Demographics of Former University of Notre Dame (ND) Players Who Only Played Through College Compared with Those Who Went on to Play Professional Football[a]

	ND College-Only N=183	ND Professional N=33	*P* value	FDR Adjusted[b]
**Age, y, median (IQR)**	67 (64, 70)	66 (63, 71)	0.90	0.90
**Race, White, n (%)**	173 (95)	28 (85)	0.06	0.20
**Years of Education, n (%)**			0.26	0.44
16, y	72 (39)	17 (52)		
≥17, y	111 (61)	16 (48)		
**Highest Level of Education, n (%)**			0.05	0.20
Bachelor's degree	90 (49)	23 (70)		
Master's degree	48 (26)	3 (9.1)		
Doctoral/Professional degree	45 (25)	7 (21)		
**Married/Domestic Partnership, n (%)**	153 (84)	27 (87)	0.82	0.90
**Employment Status, n (%)**			0.66	0.82
Full/Part-time	105 (57)	18 (55)		
Retired	75 (41)	14 (42)		
Disabled	3 (2)	1 (3)		
**Living Situation, n (%)**			0.12	0.29
Home Owner	172 (94)	28 (85)		
Renter	7 (4)	3 (9)		
Other	4 (2)	2 (6)		
**Senior Year BMI, median (IQR)**	27.4 (25.8, 29.5)	28.4 (26.4, 29.7)	0.31	0.45
**Position Played, n (%)[c]**			**0.01**	0.05
Lineman/Linebacker	95 (52)	20 (61)		
Back/Receiver	75 (41)	8 (24)		
Quarterback	4 (2)	5 (15)		
Kicker	9 (5)	0 (0)		

[a]Data reported for only living ND players who completed the survey.
[b]FDR = False discovery rate correction for multiple testing
[c]Lineman/Linebacker and Back/Receiver groups include both offensive and defensive positions.
Note: Statistics presented: n (%); median (IQR), Statistical tests performed: chi-square test of independence; Fisher's exact test. BMI = body mass index; FDR = False discovery rate; IQR = Interquartile range; ND = University of Notre Dame

© 2022 Phelps A et al. *JAMA Network Open*.

eTable 5. Health Outcomes: Former University of Notre Dame (ND) Players Who Only Played Through College Compared with Those Who Went on to Play Professional Football[a]

	ND College-Only N=183	ND Professional N=33	*P* value	FDR Adjusted[b]
**Neurological disorders, n (%)**				
Cognitive impairment diagnosis	5 (3)	5 (15)	**0.01**	0.13
Parkinson's disease	1 (1)	0 (0)	>0.99	>0.99
Recurrent headaches	17 (9)	5 (15)	0.35	0.93
**Vascular conditions, n (%)**				
Cardiovascular disease	61 (34)	9 (28)	0.66	>0.99
Heart attack history	11 (6)	0 (0)	0.22	0.77
Abnormal heart rhythm	36 (20)	8 (25)	0.67	>0.99
Artery blockage	32 (17)	1 (3)	0.06	0.46
Valve problem	9 (5)	0 (0)	0.36	0.93
Heart failure	6 (3)	1 (3)	>0.99	>0.99
Heart surgery	30 (16)	0 (0)	**0.01**	0.13
Cardiovascular risk factors	130 (72)	20 (61)	0.26	0.80
High blood pressure	91 (50)	11 (33)	0.11	0.69
High cholesterol	95 (53)	16 (48)	0.79	>0.99
Diabetes	21 (12)	3 (9)	>0.99	>0.99
Stroke	4 (2)	2 (6)	0.23	0.77
**Psychiatric diagnosis, n (%)**	27 (15)	8 (26)	0.23	0.77
Depression	22 (12)	8 (26)	0.05	0.46
Anxiety	14 (8)	3 (9)	0.73	>0.99
Bipolar disorder	0 (0)	1 (3)	0.15	0.76
Psychosis/Schizophrenia	0 (0)	0 (0)	>0.99	>0.99
**Neurodevelopmental diagnosis, n (%)**				
ADHD	5 (3)	1 (3)	>0.99	>0.99
Dyslexia	4 (2)	1 (3)	0.56	>0.99
**Sleep diagnosis, n (%)**				
Sleep apnea	49 (27)	9 (27)	>0.99	>0.99
Restless leg syndrome	2 (1)	0 (0)	>0.99	>0.99
REM sleep behavior disorder	4 (2)	1 (3)	0.58	>0.99
**Orthopedic, n (%)**				
Joint replacement	60 (33)	13 (39)	0.59	>0.99
Back surgery	21 (11)	3 (9)	>0.99	>0.99

© 2022 Phelps A et al. *JAMA Network Open*.

	Other orthopedic surgery	107 (59)	20 (62)	0.87	>0.99
**Other medical, n (%)**					
	Cancer[c]	34 (20)	1 (3)	**0.04**	0.45
	Lung condition	14 (8)	3 (9)	0.73	>0.99
	Liver disease	6 (3)	2 (6)	0.35	0.93
	Kidney disease	10 (6)	2 (6)	>0.99	>0.99
	Gastrointestinal disorder	22 (12)	6 (18)	0.40	0.94
	Eye disorder	41 (23)	6 (18)	0.70	>0.99
	Thyroid condition	23 (13)	4 (12)	>0.99	>0.99
	Low testosterone	13 (7)	2 (6)	>0.99	>0.99
**Alcohol use, n (%)**		158 (86)	27 (82)	0.59	>0.99
	Frequency among users			0.41	0.94
	Monthly or less	20 (13)	5 (19)		
	2-4 times a month	36 (23)	9 (33)		
	2-3 times a week	48 (30)	7 (26)		
	4+ times a week	54 (34)	6 (22)		
	Number of drinks when drinking			>0.99	>0.99
	1 or 2	117 (75)	20 (74)		
	3 or 4	34 (22)	6 (22)		
	5+	6 (4)	1 (4)		
**Drug use, n (%)**					
	Illicit drug use	90 (50)	14 (44)	0.67	>0.99
	Prescription drug abuse	11 (6)	0 (0)	0.22	0.77
	Performance enhancing drugs	5 (3)	5 (16)	**0.01**	0.13
**Satisfaction with Life, median (IQR)[d]**		30 (27, 33)	28 (24, 32)	0.13	0.69

[a]Data reported for only living ND players who completed the survey
[b]FDR = False discovery rate correction for multiple testing
[c]Does not include non-melanoma skin cancer.
[d]Based on the Satisfaction with Life Scale[3]
Note: Statistics presented: n (%); median (IQR), Statistical tests performed: chi-square test of independence; Fisher's exact test.
ADHD = Attention deficit hyperactivity disorder; FDR = False discovery rate; IQR = Interquartile range; ND = University of Notre Dame

© 2022 Phelps A et al. *JAMA Network Open*.

eTable 6. Health Outcomes: Former University of Notre Dame (ND) Players by Position[a]

	Lineman or Linebacker N=115	Back or Receiver N=83	Quarterback N=9	Kicker N=9	*P* value	FDR Adjusted[b]
**Neurological Disorders, n (%)**						
Cognitive impairment diagnosis	7 (6)	3 (4)	0 (0)	0 (0)	0.41	0.94
Parkinson's disease	0 (0)	0 (0)	0 (0)	1 (11)	0.08	0.68
Recurrent headaches	11 (10)	9 (11)	2 (22)	0 (0)	0.49	0.94
**Vascular Conditions, n (%)**						
Cardiovascular disease	38 (33)	27 (34)	2 (22)	3 (33)	0.96	>0.99
Heart attack history	3 (3)	8 (10)	0 (0)	0 (0)	0.19	0.79
Abnormal heart rhythm	30 (26)	13 (16)	0 (0)	1 (11)	0.15	0.79
Artery blockage	14 (12)	16 (19)	2 (22)	1 (11)	0.43	0.94
Valve problem	5 (4)	3 (4)	0 (0)	1 (11)	0.63	>0.99
Heart failure	3 (3)	4 (5)	0 (0)	0 (0)	0.71	>0.99
Heart surgery	10 (9)	19 (23)	1 (11)	0 (0)	0.03	0.67
Cardiovascular risk factors	77 (68)	63 (76)	6 (67)	4 (50)	0.34	0.94
High blood pressure	61 (54)	34 (41)	3 (33)	4 (44)	0.24	0.88
High cholesterol	50 (45)	53 (64)	4 (44)	4 (44)	0.05	0.67
Diabetes	13 (11)	10 (12)	1 (11)	0 (0)	0.94	>0.99
Stroke	3 (3)	3 (4)	0 (0)	0 (0)	0.82	>0.99
**Psychiatric diagnosis, n (%)**	21 (19)	14 (17)	0 (0)	0 (0)	0.41	0.94
Depression	19 (17)	11 (13)	0 (0)	0 (0)	0.47	0.94
Anxiety	9 (8)	8 (10)	0 (0)	0 (0)	0.96	>0.99
Bipolar disorder	1 (1)	0 (0)	0 (0)	0 (0)	>0.99	>0.99
Psychosis/Schizophrenia	0 (0)	0 (0)	0 (0)	0 (0)	>0.99	>0.99
**Neurodevelopmental diagnosis, n (%)**						
ADHD	4 (4)	2 (3)	0 (0)	0 (0)	>0.99	>0.99
Dyslexia	5 (4)	0 (0)	0 (0)	0 (0)	0.20	0.79
**Sleep diagnosis, n (%)**						
Sleep apnea	32 (28)	23 (28)	0 (0)	3 (33)	0.30	0.94
Restless leg syndrome	1 (1)	1 (1)	0 (0)	0 (0)	>0.99	>0.99
REM sleep behavior disorder	2 (2)	2 (3)	0 (0)	1 (12)	0.29	0.94
**Orthopedic, n (%)**						
Joint replacement	42 (37)	28 (34)	1 (11)	2 (22)	0.45	0.94
Back surgery	17 (15)	7 (8)	0 (0)	0 (0)	0.34	0.94

© 2022 Phelps A et al. *JAMA Network Open*.

Other orthopedic surgery	64 (57)	52 (63)	5 (62)	6 (67)	0.83	>0.99
**Other medical, n (%)**						
Cancer[c]	18 (17)	15 (19)	0 (0)	2 (25)	0.54	>0.99
Lung condition	9 (8)	6 (7)	1 (11)	1 (11)	0.74	>0.99
Liver disease	5 (4)	3 (4)	0 (0)	0 (0)	>0.99	>0.99
Kidney disease	5 (4)	6 (7)	1 (11)	0 (0)	0.46	0.94
Gastrointestinal disorder	14 (12)	9 (11)	3 (33)	2 (22)	0.16	0.79
Eye disorder	32 (28)	12 (15)	0 (0)	3 (33)	0.04	0.67
Thyroid condition	14 (12)	10 (12)	2 (22)	1 (11)	0.80	>0.99
Low testosterone	8 (7)	4 (5)	0 (0)	3 (33)	0.06	0.67
**Alcohol use, n (%)**						
Frequency among users					0.77	>0.99
Monthly or less	17 (17)	7 (10)	0 (0)	1 (12)		
2-4 times a month	24 (24)	18 (26)	2 (29)	1 (12)		
2-3 times a week	26 (2)	22 (32)	4 (57)	3 (38)		
4+ times a week	34 (34%)	22 (32)	1 (14)	3 (38)		
Number of drinks when drinking					0.09	0.68
1 or 2	69 (69)	58 (84)	5 (71)	5 (62)		
3 or 4	25 (25)	11 (16)	2 (29)	2 (25)		
5+	6 (6)	0 (0)	0 (0)	1 (12)		
**Drug use, n (%)**						
Illicit drug use	50 (44)	44 (54)	4 (44)	6 (67)	0.41	0.94
Prescription drug abuse	7 (6)	4 (5)	0 (0)	0 (0)	0.91	>0.99
Performance enhancing drugs	5 (4)	4 (5)	1 (11)	0 (0)	0.73	>0.99
**Satisfaction with Life, median (IQR)**[d]	31 (26, 33)	30 (26, 32)	34 (30, 35)	29 (28, 33)	0.16	0.79

[a]Data reported for only living ND players who completed the survey
[b]FDR = False discovery rate correction for multiple testing
[c]Does not include non-melanoma skin cancer.
[d]Based on the Satisfaction with Life Scale[3]
ADHD = Attention deficit hyperactivity disorder; FDR = False discovery rate; IQR = Interquartile range; ND = University of Notre Dame

© 2022 Phelps A et al. *JAMA Network Open*.

eTable 7. Standardized Mortality Ratios for Former University of Notre Dame (ND) Players Who Only Played Through College Compared with Other Studies of Former National Football League (NFL) Players[a]

	ND College-Only		Lincoln et. al (2018)[4]		Lehman et. al (2012)[5]	
**Cause of death**	**N**	**SMR (95% CI)**	**N**	**SMR (95% CI)**	**N**	**SMR (95% CI)**
All-cause	55	0.51 (0.39-0.67)	227	0.46 (0.40-0.52)	334	0.53 (0.48–0.59)
All cancers	16	0.60 (0.34-0.97)	23	0.41 (0.26-0.62)	85	0.58 (0.46–0.72)
Benign and unspecified nature neoplasms[b]	0	0.00 (0.00-11.45)	1	0.75 (0.02-4.16)	--	--
Diseases of blood and blood-forming organs[b]	0	0.00 (0.00-7.89)	1	0.34 (0.01-1.88)	--	--
Diabetes mellitus[b]	2	0.57 (0.07-2.07)	4	0.38 (0.10-0.96)	--	--
Mental, psychoneurotic, and personality disorders[b]	1	0.46 (0.01-2.58)	2	0.23 (0.03-0.82)	--	--
Diseases of the nervous system and sense organs[b]	2	0.96 (0.12-3.45)	5	0.65 (0.21-1.53)	--	--
Neurodegenerative causes[b,c]	2	1.22 (0.15-4.41)	--	--	10	2.83 (1.36–5.21)
Alzheimer’s disease[b]	0	0.00 (0.00-5.14)	--	--	2	1.80 (0.22–6.50)
Amyotrophic lateral sclerosis[b]	1	1.85 (0.05-10.31)	--	--	6	4.04 (1.48-8.79)
Parkinson’s disease[b]	1	2.63 (0.07-14.63)	--	--	2	2.14 (0.26–7.75)
Other diseases of the nervous system and sense organs[b]	0	0.00 (0.00-8.31)	--	--	--	--
Diseases of the heart	16	0.69 (0.39-1.12)	47	0.68 (0.50-0.90)	126	0.68 (0.56–0.81)
Hypertension w/heart disease[b]	1	0.49 (0.01-2.73)	8	0.87 (0.38-1.71)	--	--
Ischemic heart disease[b]	12	0.74 (0.38-1.30)	22	0.58 (0.36-0.87)	--	--
Cardiomyopathy[b]	0	0.00 (0.00-2.43)	7	0.75 (0.30-1.54)	--	--
Conduction disorder[b]	2	1.34 (0.16-4.86)	3	0.63 (0.13-1.84)	--	--
Other heart disease[b]	1	0.64 (0.02-3.55)	7	1.17 (0.47-2.41)	--	--
Other diseases of the circulatory system[b]	2	0.31 (0.04-1.13)	12	0.56 (0.29-0.98)	--	--
Cerebrovascular disease[b]	2	0.52 (0.06-1.88)	6	0.53 (0.19-1.14)	--	--
Diseases of the arteries, veins, and lymphatic vessels[b]	0	0.00 (0.00-2.19)	6	0.85 (0.31-1.85)	--	--
Diseases of the respiratory system[b]	0	0.00 (0.0-0.61)	2	0.15 (0.02-0.53)	--	--
Diseases of the digestive system[b]	0	0.00 (0.00-0.65)	8	0.41 (0.18-0.80)	--	--
Diseases of musculoskeletal and connective tissue[b]	0	0.00 (0.00-11.24)	1	0.61 (0.01-3.42)	--	--
Diseases of the genitourinary system[b]	0	0.00 (0.00-1.93)	1	0.17 (0.00-0.96)	--	--
Symptoms and ill-defined conditions[b]	0	0.00 (0.00-2.24)	7	0.64 (0.26-1.32)	--	--
Transportation injuries[b]	1	0.19 (0.00-1.08)	34	0.65 (0.45-0.91)	--	--
Falls[b]	0	0.00 (0.00-4.55)	2	0.58 (0.07-2.10)	--	--

© 2022 Phelps A et al. *JAMA Network Open.*

Other injuries	1	0.21 (0.01-1.17)	24	0.53 (0.34-0.79)	41	0.63 (0.45–0.86)
Drowning[b]	1	1.93 (0.05-10.75)	2	0.45 (0.05-1.61)	--	--
Forces of nature[b]	0	0.00 (0.00-23.40)	3	2.74 (0.56-8.00)	--	--
Accidental poisoning[b]	0	0.00 (0.00-2.01)	15	0.64 (0.36-1.06)	--	--
Other injury undetermined intent[b]	0	0.00 (0.00-6.46)	2	0.36 (0.04-1.29)	--	--
Other injuries[b]	0	0.00 (0.00-7.41)	2	0.55 (0.07-1.99)	--	--
Violence	1	0.14 (0.00-0.76)	39	0.38 (0.27-0.52)	13	0.27 (0.14–0.46)
Intentional self-harm[b]	1	0.29 (0.01-1.64)	20	0.58 (0.35-0.90)	--	--
Assault and homicide[b]	0	0.00 (0.00-0.93)	19	0.28 (0.17-0.43)	--	--
Other and unspecified causes	1	0.22 (0.01-1.25)	14	0.69 (0.38-1.16)	59	0.34 (0.26–0.43)
Unknown[b]	12	Not applicable[d]	--	--	--	--

[a]Standardized mortality ratios (SMRs) were calculated based on age, sex, and race with the Life Table Analysis System46 (LTAS.NET version 4.5.0) using U.S. male mortality rates (1960-2014) for 119 cause of death categories. Only Underlying Cause SMRs are presented. Multiple Cause data were also assessed and did not meaningfully change the results. Total University of Notre Dame cohort (regardless of living status or survey completion): N = 447.
[b]Data not available in all studies.
[c]SMRs for three neurodegenerative causes of death assessed with updated custom rate files utilized in previous studies.[29,48]
[d]We were unable to locate causes of death for a subset of individuals. The LTAS.NET[46] software does not calculate SMRs for “Unknown” causes of death.
CI = Confidence internal; ND = Notre Dame; SMR = Standard mortality ratio

© 2022 Phelps A et al. *JAMA Network Open*.

Supplemental References

1. NCAA sports sponsorship and participation rates database [data visualization dashboard]. National Collegiate Athletic Association. https://www.ncaa.org/about/resources/research/ncaa-sports-sponsorship-and-participation-rates-database. Updated 2020. Accessed January 10, 2021.

2. Health and Retirement Study. *2016 core survey.* Ann Arbor, MI: Health and Retirement Study, University of Michigan with funding from the National Institute on Aging (grant number NIA U01AG009740); 2016.

3. Diener E, Emmons RA, Larsen RJ, Griffin S. The Satisfaction with Life Scale. *J Pers Assess*. 1985;49:71-75.

4. Lincoln AE, Vogel RA, Allen TW, et al. Risk and causes of death among former National Football League players (1986-2012). Med Sci Sports Exerc. 2018;50(3):486-493. doi: 10.1249/MSS.0000000000001466

5. Lehman EJ, Hein MJ, Baron SL, Gersic CM. Neurodegenerative causes of death among retired National Football League players. Neurology. 2012;79(19):1970-1974. doi: 10.1212/WNL.0b013e31826daf50

© 2022 Phelps A et al. *JAMA Network Open*.
